# Benzo[1,2-b:6,5-b’]dithiophene-4,5-diamine: A New Fluorescent Probe for the High-Sensitivity and Real-Time Visual Monitoring of Phosgene

**DOI:** 10.3390/s25020407

**Published:** 2025-01-11

**Authors:** Yingzhen Zhang, Jun Xiao, Ruiying Peng, Xueliang Feng, Haimei Mao, Kunming Liu, Zhenzhong Liu, Chunxin Ma

**Affiliations:** 1 School of Chemistry and Chemical Engineering, Jiangxi University of Science and Technology, Ganzhou 341000, China; m15779018433@163.com (Y.Z.); 17679326645@163.com (J.X.); m19324876521@163.com (R.P.); 2 State Key Laboratory of Marine Resource Utilization in South China Sea, School of Chemistry and Chemical Engineering, Hainan University, Haikou 570228, China; 22220856010033@hainanu.edu.cn; 3 Key Laboratory of Quality Safe Evaluation and Research of Degradable Material, State Administration for Market Regulation, Hainan Academy of Inspection and Testing, Haikou 570203, China; mcmhm@163.com; 4 Taizhou Key Laboratory of Medical Devices and Advanced Materials, Taizhou Institute of Zhejiang University, Taizhou 318000, China; zzliu@zju.edu.cn

**Keywords:** fluorescent chemosensors, phosgene, benzo[1,2-b:4,3-b’]dithiophene, real-time monitoring, visual test strip

## Abstract

The detection of highly toxic chemicals such as phosgene is crucial for addressing the severe threats to human health and public safety posed by terrorist attacks and industrial mishaps. However, timely and precise monitoring of phosgene at a low cost remains a significant challenge. This work is the first to report a novel fluorescent system based on the Intramolecular Charge Transfer (ICT) effect, which can rapidly detect phosgene in both solution and gas phases with high sensitivity by integrating a benzo[1,2-b:6,5-b’]dithiophene-4,5-diamine (BDTA) probe. Among existing detecting methods, this fluorescent system stands out as it can respond to phosgene within a mere 30 s and has a detection limit as low as 0.16 μM in solution. Furthermore, the sensing mechanism was rigorously validated through high-resolution mass spectrometry (HRMS) and density functional theory (DFT) calculations. As a result, this fluorescent probing system for phosgene can be effectively adapted for real-time, high-sensitivity sensing and used as a test strip for visual monitoring without the need for specific equipment, which will also provide a new strategy for the fluorescent detection of other toxic materials.

## 1. Introduction

Phosgene, chemically known as carbonyl dichloride, is one of most important industrial raw materials extensively utilized in polymers, pharmaceuticals, pesticides, dyes, and so on [1]. Although phosgene plays a critical role in the production of carbonates, polycarbonates, isocyanates, acyl chlorides, anhydrides, and heterocyclic compounds [2,3], it is very dangerous due to the high toxicity, and was exploited during World War I for the creation of chemical warfare agents (CWAs) [4]. Exposure to phosgene can be perilous, as the gas can penetrate the lower respiratory tract, leading to lung damage and alveolar collapse, which in turn causes severe inflammation of the respiratory tract and lung tissues [5]. Furthermore, phosgene can disrupt the oxidative stress system, affect the pulmonary vascular growth factor system, and increase the permeability of vascular endothelium, potentially leading to respiratory failure or death in severe cases. Phosgene is particularly dangerous in production, transportation, storage, and usage, due to its high toxicity, combined with being colorless and having a faint odor. Nowadays, detecting phosgene precisely and rapidly at a low cost remains a significant challenge [6]. As a consequence, developing a real-time and sensitive detection assay for phosgene is crucial for protecting public health and safety [7,8].

Recently, various analytical methods including gas chromatography, liquid chromatography, electrochemical techniques, and spectrophotometric approaches have been developed to detect trace levels of phosgene [9,10,11,12]. Among them, fluorescent sensors have garnered significant interest due to their operational simplicity, rapid response, exceptional selectivity, and sensitivity [13,14,15]. Several fluorescent probes have been engineered for phosgene detection, leveraging diverse sensing mechanisms [16,17,18,19,20,21,22].

When designing a fluorescent probe for detection, it is essential to consider a number of physical and chemical properties of the probe backbone. These include excitation and emission properties, photostability, and biofilm permeability. Over the past decades, a number of fluorophores have been employed in this field, including rhodamine [23], coumarin [24], BODIPY [25], phloroglucinol [26], and naphthylamide [27,28]. The carbonyl group of phosgene has two electronegative chlorine atoms attached to it, which renders it a strong amphiphilic potential site. The majority of fluorescent probes employed for the detection of phosgene contain electron-donating recognition groups, such as N, O, etc. Two neighboring recognition groups interact with phosgene to form a pentacyclic or six-membered ring, which results in a change in its photophysical properties [21,29,30]. Nevertheless, the integration of recognition groups with the fluorophore is predominantly achieved through chemical modification, which often presents challenges in the synthesis process and can escalate production costs.

Benzodithiophene (BDT), as the dithiophene counterpart to phenanthrene, has emerged as a preferred electron-donating unit for π-conjugated polymers [31,32]. Its expansive planar π-conjugated framework facilitates efficient π-π stacking, thereby enhancing hole mobility [33,34]. Concurrently, the planar and rigid fused aromatic structure of BDT enhances intermolecular interactions within polymer chains, which is beneficial for charge transport [35,36]. Notably, this preference is attributed to its generally lower reorganization energies and improved solubility in organic solvents, which confer significant advantages over benzene-based analogs in the context of molecular sensing [37]. Nevertheless, up to now, there have been very few examples of chemical sensors based on BDT [38], and sensors for detecting phosgene have not yet been reported.

**Scheme 1:**
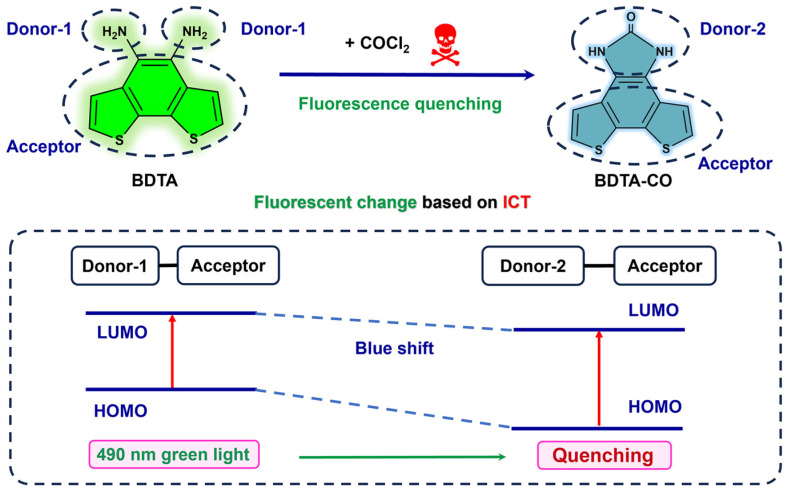
Detection principle of **BDTA** for phosgene determination.

Herein, we report a new fluorescent probe for rapidly detecting phosgene in both solution and gas with high sensitivity (Scheme 1). Based on the analysis above, we propose that this commercial benzo[1,2-b:6,5-b’]dithiophene-4,5-diamine (BDTA) can, in theory, be a probe for detecting phosgene: the BDT group and *ortho*-diamine group are linked via a conjugated system, creating an electron system with a push–pull effect that generates an Intramolecular Charge Transfer (ICT) effect. Upon reaction of the *ortho*-diamine group with phosgene to form a urea group through intramolecular cyclization, there is a decrease in electron cloud density and an increase in the energy gap between HOMO and LUMO, leading to a blue shift and consequent fluorescent quenching (Scheme 1). This rapid and precise detection of phosgene based on the BDTA has been confirmed for the first time, which can be utilized for real-time and high-sensitivity sensing and can also be used as a test strip for naked-eyed monitoring without the need for specific equipment.

## 2. Materials and Methods

### 2.1. Materials

Benzo[2,1-b:3,4-b’]dithiophene-4,5-diamine (BDTA) (Nanjing Zhiyan Technology Co. Ltd., Nanjing, China), triphosgene (BTC), CH_3_COCl (Shanghai Aladdin Biochemical Technology Co. Ltd., Shanghai, China), 4-nitrobenzenesulfonyl chloride (*p*-NsCl), CF_3_COOH, HCOOH, C_2_H_2_O_2_ (Beijing InnoChem Science & Technology Co., Ltd., Beijing, China), POCl_3_ (Jiuding Chemical Science & Technology Co., Ltd., Shanghai, China), HCl, HCHO (Xilong Scientific Co., Ltd., Guangdong, China), SO_2_Cl_2_ (Shanghai Macklin Biochemical Technology Co., Ltd., Shanghai, China), C_6_H_5_COCl (Sinopharm Chemical Reagent Co., Ltd., Shanghai, China), C_3_H_4_O (Sahn Chemical Technology Co., Ltd., Shanghai, China). Dimethylsulfoxide (DMSO) (Shanghai Macklin Biochemical Technology Co., Ltd., Shanghai, China), DMF (dimethylformamide), THF (tetrahydrofuran), and acetonitrile (ACN) (Xilong Scientific Co., Ltd., Guangdong, China) used without further purification.

### 2.2. General Instruments

Emission spectra were obtained using a fluorescence spectrometer (F-4600, Hitachi, Japan) whose error limits were estimated as follows: λ (±1 nm); τ (±10%); φ (±10%). Mass spectrometry was performed using the Bruker ESI-Q-TOF MS/MS, Bremen, German. The Fourier transform infrared radiation (FT-IR) spectra were recorded with the FT-IR (Bruker ALPHA, German) instrument by the ATR apparatus with 4 cm^−1^ resolution between 4000 and 400 cm^−1^.

The 365 nm ultraviolet (UV) resource was used to excite the fluorescence emission with the ZF-1 Ultraviolet Analysis Instrument, Hangzhou Qiwei Instrument Co., Ltd., Hangzhou, China.

### 2.3. Spectral Analysis

Triphosgene, a solid at room temperature, can undergo organic chemical reactions similar to those of phosgene and is known as solid phosgene. In order to avoid the direct use of highly toxic phosgene gas, the low toxicity triphosgene is often used in scientific research to participate in the reaction instead of phosgene. Therefore, we use triphosgene instead of phosgene for the detection of phosgene in solution. The triphosgene solution was prepared with acetonitrile, and the purchased acetonitrile reagent was used directly without further purification. A 1 mM triphosgene acetonitrile stock solution was prepared. When used, the triphosgene reserve solution was diluted to obtain acetonitrile solutions with triphosgene concentrations of 5–200 μM.

BDTA was dissolved in DMSO to form a stock solution with a concentration of 1.0 mM and stored at a cool temperature. The reserve solution of BTC was used immediately after preparation as a source of phosgene. 4-Nitrobenzenesulfonyl chloride (*p*-NsCl), 4-toluene sulfonyl chloride (TsCl), CH_3_COCl, CF_3_COOH, POCl_3_, HCl, HCHO, SO_2_Cl_2_ C_6_H_5_COCl, and HCOOH were dissolved in acetonitrile to create the required concentrations for selectivity and competition experiments. The stock solution of BDTA was diluted to a concentration of 100 μM, and various concentrations of BTC (ranging from 0 to 200 μM) were added for fluorescent titration. The resulting mixture was then allowed to react at room temperature for 30 s. Subsequently, the fluorescence intensity was measured. The excitation wavelength was set to 273 nm, and the emission spectrum was scanned from 293 nm to 800 nm, with both excitation and emission slit widths set to 10 nm.

The experimental details on dissolution screening are provided in the ESI file. As for sensitivity testing, BDTA (100 μM) and various concentrations of BTC (0–200 μM) were added into a cuvette sequentially. After the above mixed solutions were reacted for 30 s, a fluorescence spectrophotometer was used.

The limit of detection (LOD) was calculated according to the following formula:

LOD=3σ/k

where σ is the standard deviation of blank measurement, and *k* is the slope between the fluorescence intensity versus the triphosgene concentration.

### 2.4. Mechanism Validation and Application

BDTA (100 μM) and BTC (100 μM) were combined in a 500 μL volume and reacted fully, and the resulting products were identified by high-resolution mass spectrometry. For the preparation of test strips, the test strips were fully immersed in 10 mM BDTA solution for 5 min and 10 mM BTC solution was dropped on the test strips using a capillary tube. In the detection of gaseous phosgene, the corresponding phosgene concentration was obtained by mixing triphosgene with triethylamine (3 eq). The test paper was cut into small disks with a radius of 1 cm and soaked in a solution containing 10 mM BDTA for 5 min. The disks were then placed in a glass bottle with a lid, and different concentrations of phosgene were added to the bottle. The bottle was covered and left to stand for 5 min to observe the phenomenon.

### 2.5. Theoretical Quantum Calculations to Explain the Change In Fluorescent Emission

Density functional theory (DFT) and Time-Dependent Density Functional Theory (TDDFT) calculations were performed to obtain the lowest unoccupied molecular orbitals (LUMOs) and the highest occupied molecular orbitals (HOMOs) of compounds. The optimisation of the matter and the calculations of the energy levels were carried out using Gaussian 16W and with B3LYP/6-31G(d,p) and SCRF = (smd, solvent = DMSO) [39]. All structures calculated were geometry- and frequency-optimized using Gaussian 16.0 supported by Gauss View 6.0.

## 3. Results and Discussion

**Figure 1:**
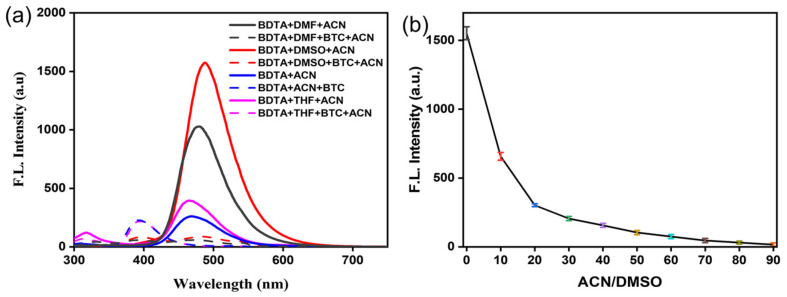
(**a**) Fluorescence emission spectra of the sensing system containing BDTA (100 μM), with or without BTC (200 μM) in indicated solvents; (**b**) the effect of ACN content on the probes under DMSO-ACN (λem = 490 nm, slits: 10/10 nm), rt for 30 s.

Due to electrostatic interactions between solute and solvent molecules in solution, different electron distributions result in different dipole distances and polarizations of the ground and excited states of solute molecules. This causes the ground and excited states to interact with the solvent molecules to varying degrees, which greatly affects the fluorescence emission and intensity. Hence, we first studied the influence of BDTA on BTC recognition in different solvent systems (Figure 1a). Common organic solvents such as tetrahydrofuran (THF), acetonitrile (ACN), N, N-dimethylformamide (DMF), and dimethylsulfoxide (DMSO) were selected as detection solutions, and the fluorescence spectra of BDTA before and after adding BTC were measured. The solutions of BDTA alone exhibited strong fluorescence emission above 490 nm in each solution, and a 15-fold fluorescence decrease could be observed in DMSO upon addition of BTC, while only slight blue shifts occurred in acetonitrile or THF, which indicates the relatively significant solvent effect in the sensing system. However, it was found in the detection system that a 16-fold decrease in fluorescence intensity in the system using DMSO-ACN was helpful for the accurate determination of triphosgene. Furthermore, utilizing the Uv-vis and fluorescence spectra as a foundation, the excitation wavelength was set to 273 nm (Figure S1). Therefore, DMSO-ACN was chosen as the detection solvent in subsequent experiments. Considering the poor solubility of the monomer in ACN, the DMSO-ACN ratio of the detection system was optimized (Figure 1b). At room temperature, it was found that the fluorescence intensity decreased with the increase in ACN content in the solvent, and the greatest decrease was observed when DMSO:ACN = 1:9, *v*/*v*.

**Figure 2:**
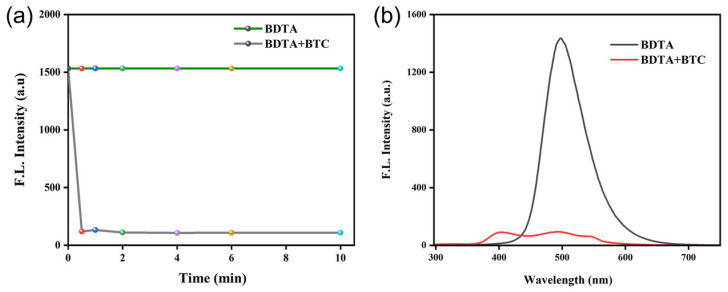
(**a**) The fluorescence intensity of the system in the presence and absence of BTC in ACN at the indicated time. (**b**) Fluorescence emission spectra of BDTA in DMSO before and after the addition of BTC at the incubation time of 30 s. The detection concentration is 100 μM for BDTA in DMSO and 200 μM for BTC in ACN (λem = 490 nm, slits: 10/10 nm).

Incubation time is an important indicator for evaluating the response ability of probes. Considering the importance of response time in the sensing system, we examined the alterations in fluorescence at different incubation times. As depicted in Figure 2, BDTA exhibited a consistent fluorescence intensity over an extended period. However, upon the addition of triphosgene, there was a rapid decrease in fluorescence intensity within 30 s, followed by a subsequent stabilization. This suggests that the alteration in fluorescence intensity of the detection system is directly attributable to the presence and action of triphosgene. To ensure a stable fluorescence signal for subsequent fluorescence titration experiments, 0.5 min was selected as the optimal response time.

**Figure 3:**
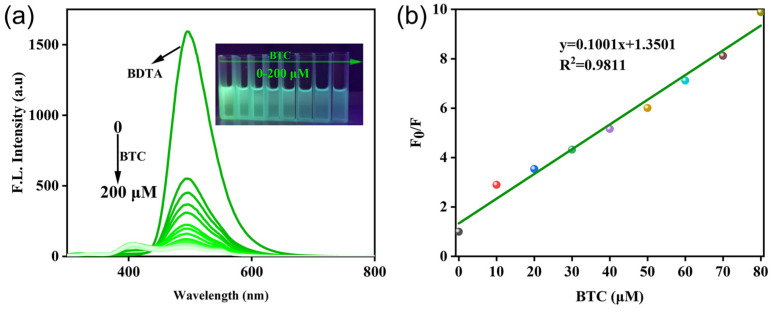
(**a**) Fluorescence spectra of BDTA (100 μM) in response to different concentrations of BTC (0–200 μM), inset: photograph of 100 μM of BDTA with 0–200 μM of BTC 365 nm UV light; (**b**) linear relationship between BDTA and BTC (λem = 490 nm, slits: 10/10 nm).

To evaluate sensitivity, fluorescence titration experiments were conducted using various concentrations of triphosgene. When the concentration of triphosgene was increased from 0 to 200 μM, the fluorescence emission intensity decreased significantly, and the fluorescence intensity was reduced by a factor of 16 (Figure 3a). In addition, there was a significant change in the concentration of triphosgene at 0–200 μM under 365 nm UV light (Figure 3a, inset). Figure 3b reveals a good linear correlation between the fluorescence intensity at 490 nm and the concentration of triphosgene between 0 and 80 μM, and the limit of detection was calculated to be 0.16 μM according to LOD = 3σ/*k* (where σ is the standard deviation of blank measurement, and *k* is the slope between the fluorescence intensity versus triphosgene concentration). According to Table 1, it can be seen that the probe in this work has high sensitivity to phosgene in the DMSO-ACN solution, and this system probe has some practicality in practical phosgene detection.

**Figure 4:**
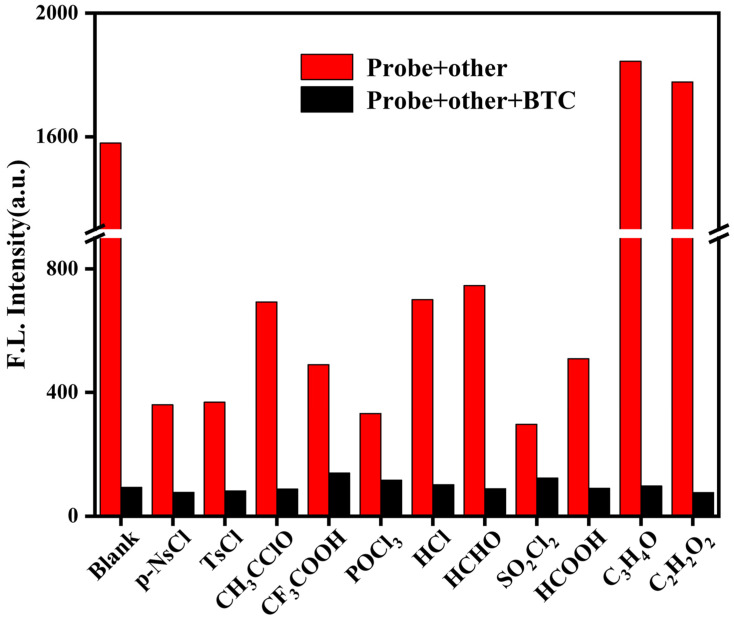
BDTA (100 μM) with various interfering substances (200 μM) and with or without BTC fluorescence response (λem = 490 nm, slits: 10/10 nm).

The high selectivity of the probes for the target analyte over potential interference is one of the most important bases for their application in a practical setting. The presence of numerous toxic substances, which differ in structure and pathogenic mechanism from phosgene, highlights the need for phosgene-specific detection. As illustrated in Figure 4 and Figure S2, the addition of 11 common toxic substances to BDTA resulted in only minimal and insignificant fluorescence quenching. However, upon the introduction of triphosgene into the detection system, a pronounced and significant decrease in fluorescence intensity was observed. This indicates that the probe can achieve selective recognition of phosgene among many interfering substances.

**Figure 5:**
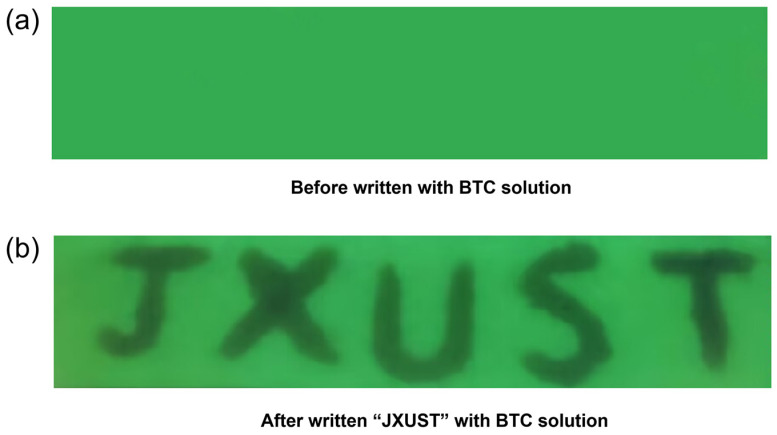
Fluorescence changes of BDTA test strip without (**a**) and with (**b**) 10 mM BTC solution.

Given the high sensitivity, selectivity, and rapid response ability of the probe BDTA for detecting phosgene in solution, we prepared a test strip based on it and tried to use it for the visual identification of phosgene. As shown in Figure 5, the test strip was fully immersed in 10 mM of BDTA solution for 5 min, and 10 mM of BTC solution was dropped onto the test strip using a capillary tube with the words “**JXUST**”. Under 365 nm UV light, the test strip fluorescence burst, and the words “**JXUST**” can be clearly seen.

**Figure 6:**
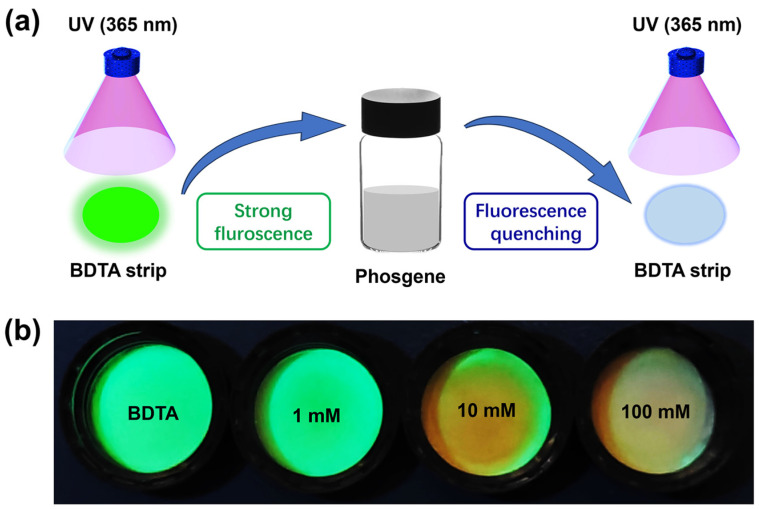
(**a**) Schematic representation of phosgene detection using test strips. (**b**) Photos of BDTA (10 mM)-loaded paper strips exposed to various concentrations of phosgene.

Due to the toxicity of phosgene, a straightforward detection method for gaseous phosgene was developed. The test strip was cut to the size of the reagent bottle cap and placed on the inside of the cap. The strip was then exposed to a certain concentration of phosgene gas to evaluate its visual recognition capability for phosgene vapor. At room temperature, the test strip responded immediately to phosgene concentrations in the range of 1 mM to 100 mM (Figure 6). With an increase in phosgene concentration, there was a progressive decrease in the test strip’s fluorescence, a change that was visibly noticeable to the naked eye. This phenomenon suggests that the test strip could serve as an effective tool for the real-time, early detection of trace amounts of phosgene in the environment.

To verify the detection mechanism of the reaction between BDTA and phosgene, we performed high-resolution mass spectrometry (HRMS) analyses to monitor the reaction mixtures. Phosgene, generated in situ by reacting triphosgene with triethylamine (3 eq), was injected into the **BDTA** solution. The molecular mass 245.99 of the new peak was confirmed by mass spectrometry (Figures S4 and S5), corresponding to the molecular weight of the reaction product ([M]: 245.99, [M + H]: 246.99). Additionally, the FT-IR spectrum indicates that, compared to BDTA, the reaction mixture exhibits a new characteristic peak at 1683 cm^−1^, suggesting the formation of the C=O bond in the urea group (Figures S6 and S7). These data indicate that the fluorescence decrease is due to the cyclization reaction to produce benzimidazolone (BDTA-CO).

**Figure 7:**
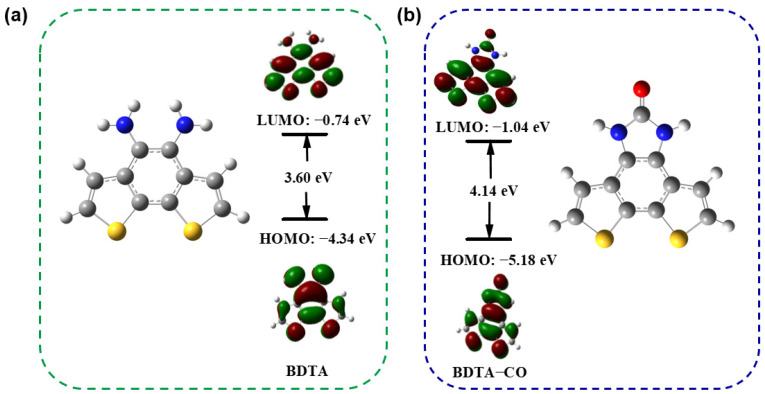
Frontier molecular orbitals of **BDTA** (**a**) and **BDTA-CO** (**b**) in DMSO by DFT at the B3LYP/6-31G.

DFT computations employing the model B3LYP and 6-31+G(d,p) basis set were conducted to elucidate the optical responses of both BDTA and BDTA-CO. The optimized ground-state structures were calculated by TDDFT (Figure S8 and Table S1), and the HOMO and LUMO of BDTA and BDTA-CO calculated by DFT are shown in Figure 7. In the case of BDTA, the electron density is distributed between the amino group and benzene ring in the HOMO, while it extends toward the thiophene in the LUMO, indicating an ICT in its excited state. Conversely, in BDTA-CO, the electrons are delocalized across the entire molecule in the HOMO. In the LUMO of BDTA-CO, the electron density is moved away from the amide moiety, showing electron deficiency in that region, which hinders the ICT process. Additionally, during the transformation from BDTA to BDTA-CO, the dihedral angle of the probe changes, and the free rotation of the amino group is suppressed, which may also be one of the reasons for the change in fluorescence. The blue shift in BDTA-CO’s emission spectra is supported by its higher HOMO-LUMO energy gap (4.14 eV) than that of BDTA (3.60 eV).

## 4. Conclusions

In summary, this study is the first to report a novel fluorescent system which can rapidly detect phosgene in both solution and gas phases with high sensitivity by integrating a commercially available benzo[1,2-b:6,5-b’]dithiophene-4,5-diamine (BDTA) probe. The BDTA responded to phosgene within only 30 s, boasting a detection limit as low as 0.16 μM in solutions, which is outstanding among existing methods. Furthermore, the sensing mechanism was rigorously validated through HRMS, FT-IR spectrum, and DFT calculations. Therefore, this probing system for phosgene can be effectively adapted for real-time and high-sensitivity sensing, and can also be used as a test strip for visual monitoring without the need for specific equipment. This work provides a new strategy for the fluorescent detection of other toxic materials based on the Intramolecular Charge Transfer (ICT) effect.

## Figures and Tables

**Table 1:** Comparison of reported probe properties.

Entry	Probe	Linear Range (μM)	LOD	Response Time	Reference
1	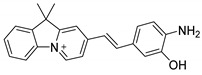	0–2.5	5.1 μM	60 s	[26]
2	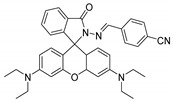	2–7	3.5 μM	30 s	[30]
3	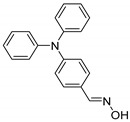	-	21.5 μM	30 s	[40]
4	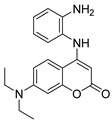	0–45	3 nM	60 s	[41]
5	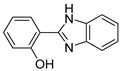	0–6	14.5 nM	20 min	[42]
6	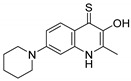	0–36	14 nM	44 s	[43]
7	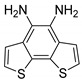	0–80	0.16 μM	30 s	This work

## Data Availability

Data are contained within the article.

## Supplementary Materials

The following supporting information can be downloaded at: https://www.mdpi.com/article/10.3390/s25020407/s1, Figure S1. The Uv-vis and fluorescence spectra within 100 μM of BDTA; Figure S2. Fluorescence spectra of the reaction of BDTA with various analytes; Figure S3. Chemical reaction equation of BDTA with phosgene; Figures S4 and S5: The mass spectra of the reaction between BDTA and BTC; Figure S6. FT-IR spectra of BDTA; Figure S7. FT-IR spectra of BDTA-CO; Figure S8. Uv-vis Spectra of BDTA and BDTA-CO (100 μ M) in DMSO; Table S1. Photophysical Data of BDTA and BDTA-CO.
