# Supplementary material for: Benzo[1,2-b:6,5-b’]dithiophene-4,5-diamine: A New Fluorescent Probe for the High-Sensitivity and Real-Time Visual Monitoring of Phosgene
Source: Sensors (Basel). 2025 Jan 11;25(2):407. doi: 10.3390/s25020407 (PMC11768993; doi:10.3390/s25020407)
Supplement: Supplementary file 1 [file sensors-25-00407-s001.zip › sensors-3395335-supplementary.pdf]

Benzo[1,2-b:6,5-b']Dithiophene-4,5-Diamine: A New Fluorescent Probe for High-Sensitive and Real-Time Visual Monitoring of Phosgene

Yingzhen Zhang [1], Jun Xiao [1], Ruiying Peng [1], Xueliang Feng[2], Haimei Mao [3], Kunming Liu [1,*], Zhenzhong Liu 4, Chunxin Ma [2, 4,*]

[1] School of Chemistry and Chemical engineering, Jiangxi University of Science and Technology, 86 Hongqi Road, Ganzhou 341000, P. R. of China

[2] State Key Laboratory of Marine Resource Utilization in South China Sea, School of Chemistry and Chemical Engineering, Hainan University, Haikou 570228, China

[3] Key Laboratory of Quality Safe Evaluation and Research of Degradable Material, State Administration for Market Regulation; Hainan Academy of Inspection and Testing, Haikou, Hainan 570203, China (H.-M. M.)

[4] Taizhou Key Laboratory of Medical Devices and Advanced Materials, Taizhou Institute of Zhejiang University, Taizhou 318000, China.

* Correspondence: liukunming@jxust.edu.cn (K.-M. L.); machunxin@hainanu.edu.cn (C.-X. M.)

1. Spectral analysis

A certain quantity of BDTA was dissolved in DMF, DMSO, THF and acetonitrile to form a 1 mM stock solution, and a certain quantity of BTC was dissolved in acetonitrile to form a 1 mM stock solution, which was stored at a low temperature. The BDTA reserve solution, dissolved in different solvents, was diluted to 100 μM, and the BTC reserve solution was diluted to 200 μM, respectively. These solutions were then mixed in a volume ratio of 1:1 at room temperature, and reacted fully for 30 s. In the optimal solvent ratio test, BDTA (μM) and BTC (200 μM) were tested in different volume ratios.

Triphosgene, a solid at room temperature, can undergo organic chemical reactions similar to those of phosgene and is known as solid phosgene. In order to avoid the direct use of highly toxic phosgene gas, the low toxicity triphosgene is often used in scientific research to participate in the reaction instead of phosgene. Therefore, we use triphosgene instead of phosgene for the detection of phosgene in solution. The triphosgene solution was prepared with acetonitrile, and the purchased acetonitrile reagent was used directly without further purification. 1 mM triphosgene acetonitrile stock solution was prepared. When used, dilute the triphosgene reserve solution, respectively, to obtain the concentration of 5-200 μM triphosgene acetonitrile solution.

BDTA was dissolved in DMSO to form a stock solution with a concentration of 1.0 mM and stored at cool temperature. The reserve solution of BTC was used immediately after preparation as a source of phosgene. 4-Nitrobenzenesulfonyl chloride (*p*-NsCl), 4-toluene sulfonyl chloride (TsCl), CH_3COCl, CF_3COOH, $POCl_3$, HCl, HCHO, SO_2Cl_2 C_6H_5COCl, C_3H_4O, $C_2H_2O_2$ and HCOOH were dissolved in acetonitrile to create the required concentrations for selectivity and competition experiments. The stock solution of BDTA was diluted to a concentration of 100 μM, and various

concentrations of BTC (ranging from 0 to 200 μM) were added for fluorescent titration. The resulting mixture was then allowed to react at room temperature for 30 s. Subsequently, the fluorescence intensity were measured. The excitation wavelength was set to 273 nm, and the emission spectrum was scanned from 293 nm to 800 nm, with both excitation and emission slit widths set to 10 nm.

The experimental details of fluorescent detecting condition optimization were provided in the ESI file. As for sensitivity testing, BDTA (100 μM) and various concentrations of BTC (0-200 μM) were added in a cuvette sequentially. After the above mixed solutions were reacted for 30 s, fluorescence spectrophotometer.

The limit of detection (LOD) is calculated according to the following formula:

$$LOD = 3\delta / K$$

where δ is the standard deviation of blank measurement, k is the slope between the fluorescence intensity versus triphosgene concentration.

2. Optical Properties of Probes

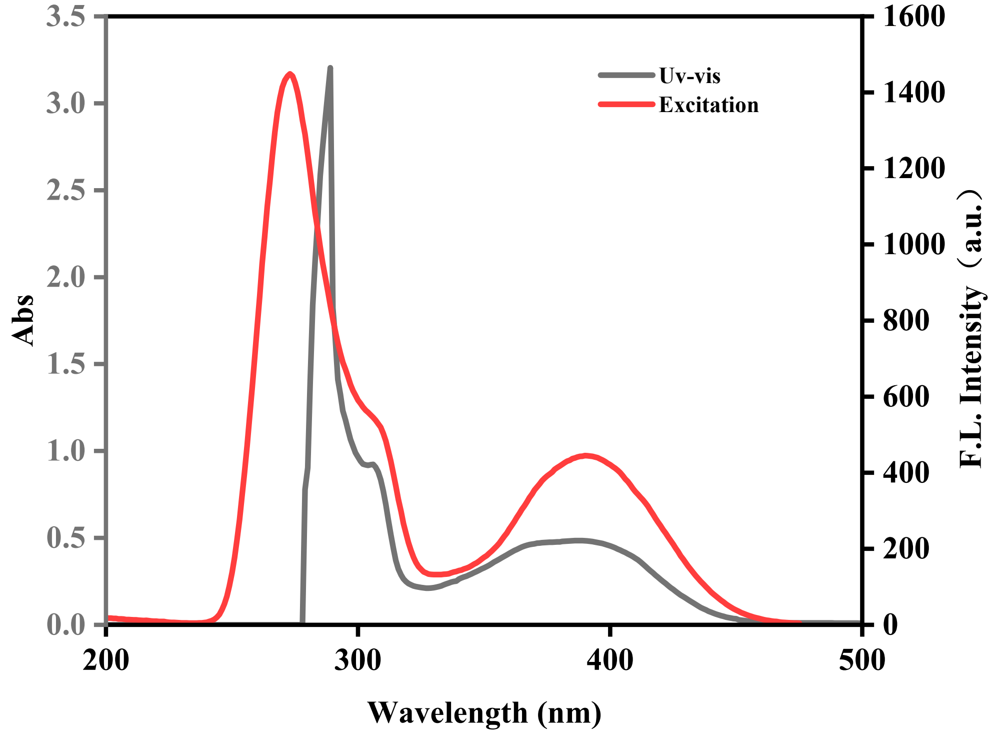

Figure S1. The Uv-vis and fluorescence spectra within 100 μM of BDTA. (λem = 490 nm, slits: 10/10 nm)

3. Selective testing of probes

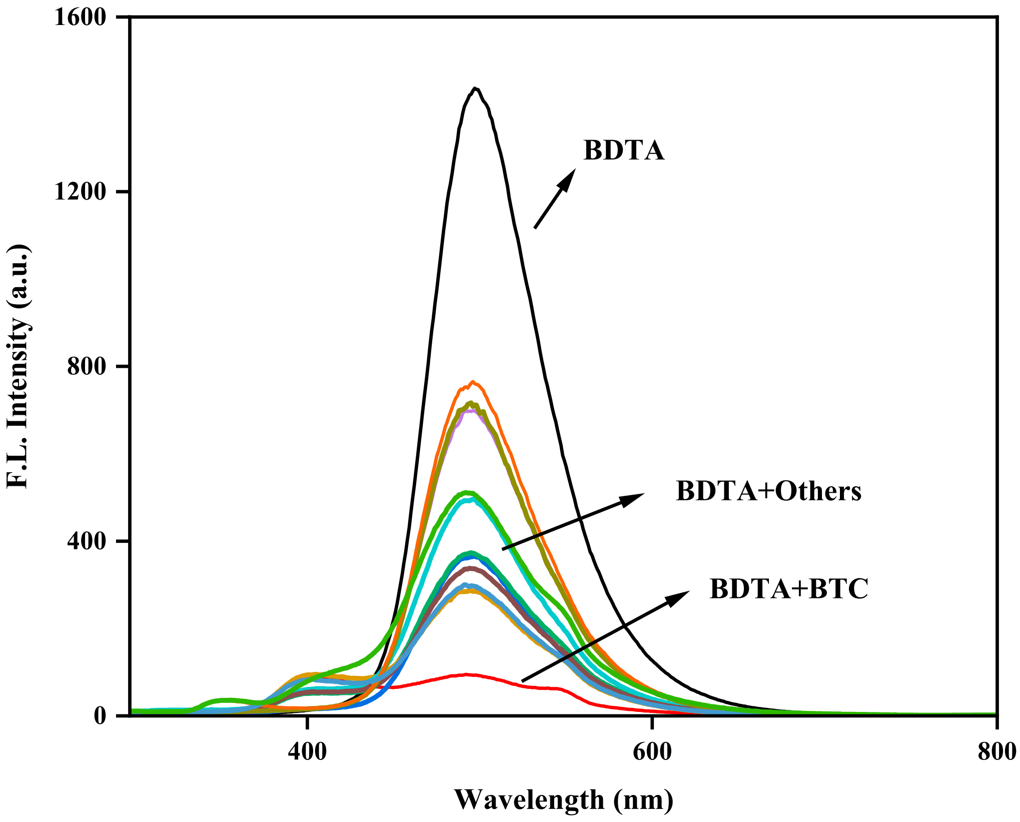

Figure S2 Fluorescence spectra of the reaction of **BDTA** with various analytes. (Othes: *p*-NsCl, TsCl, CH_3COCl, CF_3COOH, $POCl_3$, HCl, HCHO, SO_2Cl_2, C_6H_5COCl, HCOOH).

4. Mechanical study

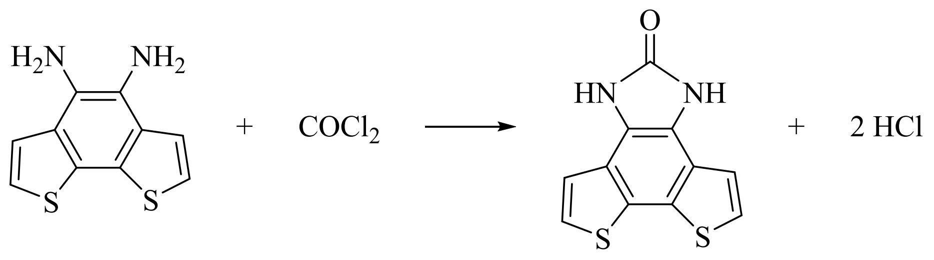

Figure S3 Chemical reaction equation of **BDTA** with phosgene.

The predicted reaction is illustrated in Figure S3. To validate the detection mechanism of the reaction between BDTA and phosgene, a mass spectrometry (MS) analysis was conducted by injecting phosgene into the BDTA solution to monitor the reaction mixture. As shown in Figure S4, the signal peaks for the unreacted **BDTA** and BTC are located at 221.9735 and 296.7551, respectively. The molecular weight of the new peak, 245.99, was confirmed by mass spectrometry (Figure S4-S5) and was determined to be the molecular weight resulting from the reaction ([M]: 245.99, [M + H]: 246.99). Furthermore, infrared spectroscopy (Figure S6-S7) was employed to examine the probe and reactants of the probe and phosgene. The FT-IR spectrum of BDTA-CO exhibited a distinctive peak at 1683 cm^{-1}, indicative of a C=O bond in urea group. These findings suggest that the observed fluorescence enhancement may be attributed to the ring-forming condensation reaction that generates the urea.

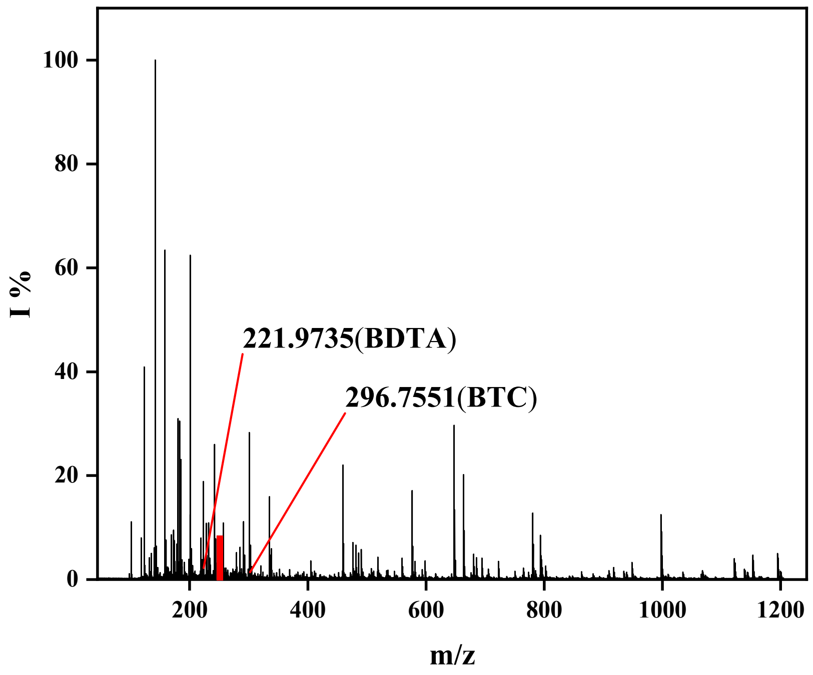

Figure S4 The HRMS of the reaction between **BDTA** and BTC (full diagram)**.**

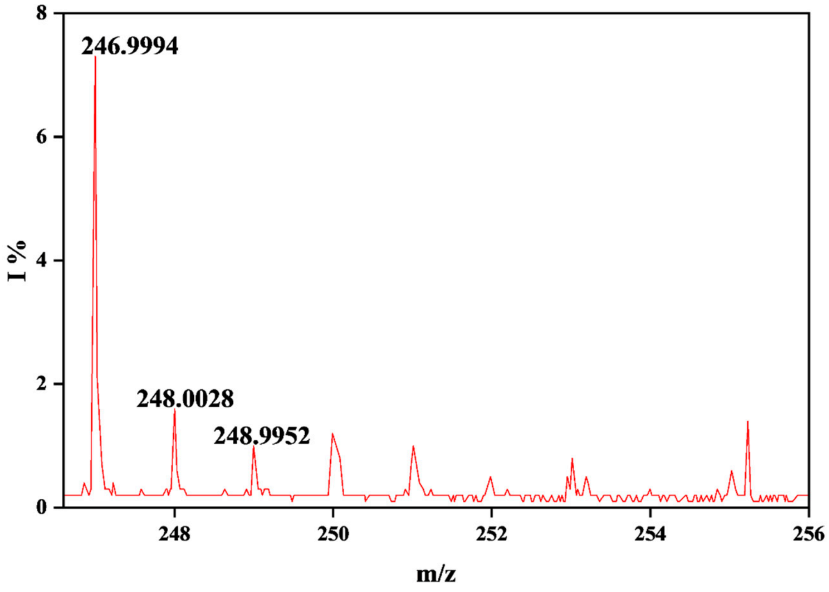

Figure S5 The HRMS of the reaction between **BDTA** and BTC (local enlargement diagram)**.**

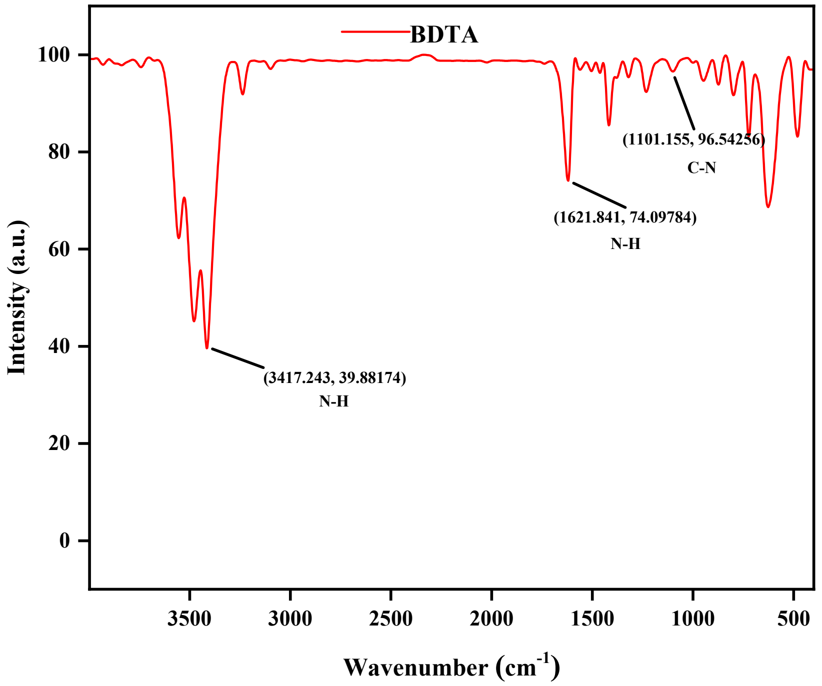

Figure S6 FT-IR spectra of **BDTA**.

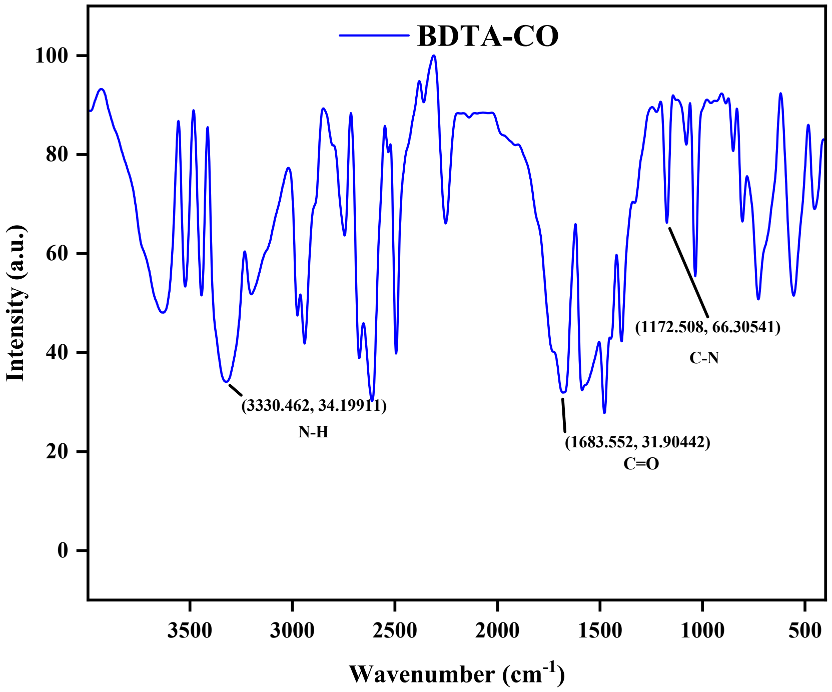

Figure S7 FT-IR spectra of **BDTA-CO**.

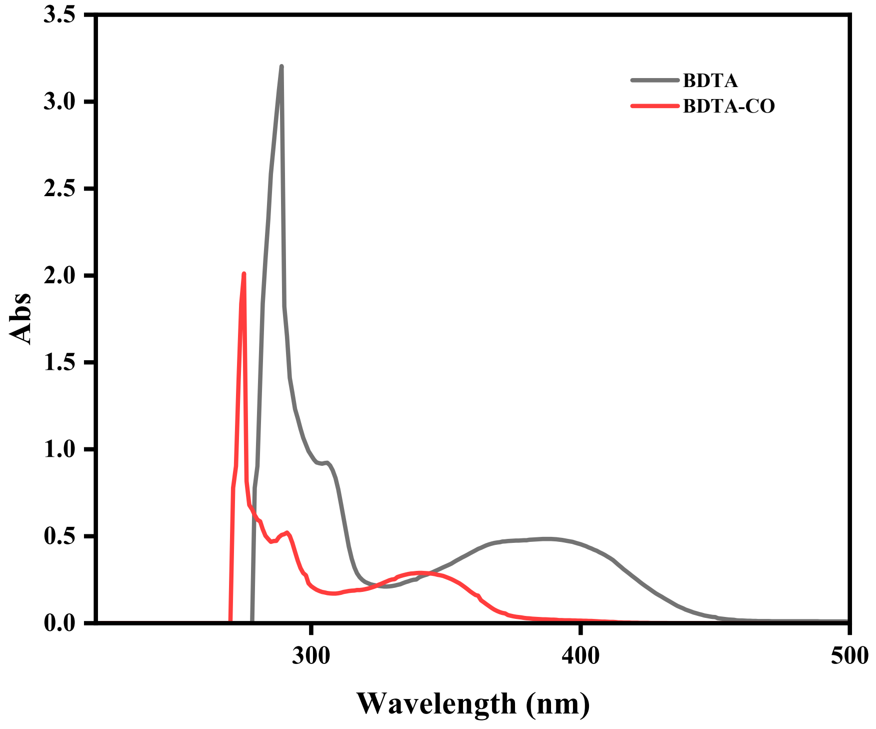

Figure S8 Uv-vis Spectra of BDTA and BDTA-CO (100 μ M) in DMSO

Table S1 Photophysical Data of BDTA and BDTA-CO

Compound	Es[a]	E(eV)/(nm)	Os[b]	Configuration	Assign
BDTA	S_1	2.777S eV / 446.42 nm	0.20150	H → L 99.0%	^{1}MLCT/^{1}LLCT
BDTA-CO	S_1	3.6234 eV / 342.18 nm	0.18380	H → L 95.4%	^{1}MLCT/^{1}LLCT

[a] Es= excited state; [b] Os = oscillator strengths

To further understand the photophysical properties, density functional theory (DFT) and (TDDFT) calculations of BDTA and BDTA-CO in DMSO media were performed. DFT and TDDFT computations employing the model B3LYP and 6-31+G (d, p) basis set were conducted to elucidate the optical responses of both BDTA and BDTA-CO. The optimized ground-state structures and the HOMO and LUMO of BDTA and BDTA-CO are shown in Fig 7, Table S1 and Figure S8. The calculated lowest-energy absorption at 446.42 nm of BDTA, corresponding to the 330-460 nm

absorption shoulder in experiment, mainly originates from the S_1 state, which is primarily composed of the HOMO → LUMO (99.0%). As shown in Figure S8 and Table S1, the calculated lowest-energy absorption at 342.18 nm of BDTA-CO, corresponding to the 300-400 nm absorption shoulder in experiment, mainly originates from the S_1 state, which is primarily composed of the HOMO → LUMO (95.4%). In the case of BDTA, the electron density is distributed between the amino group and benzene ring in the HOMO, while it extends toward the thiophene in the LUMO, indicating an ICT in its excited state. Conversely, in BDTA-CO, the electrons are delocalized across the entire molecule in the HOMO. In the LUMO of BDTA-CO, the electron density is moved away from the amide moiety showing electron deficiency in that region, which hinders the ICT process. The blue shift in BDTA-CO's emission spectra is supported by its higher HOMO-LUMO energy gap (4.14 eV) than that of BDTA (3.60 eV).
